# Binding and Functional Folding (BFF): A Physiological Framework for Studying Biomolecular Interactions and Allostery

**DOI:** 10.1016/j.jmb.2022.167872

**Published:** 2022-10-28

**Authors:** Brianna D. Young, Mary E. Cook, Brianna K. Costabile, Riya Samanta, Xinhao Zhuang, Spiridon E. Sevdalis, Kristen M. Varney, Filippo Mancia, Silvina Matysiak, Eaton Lattman, David J. Weber

**Affiliations:** 1 The Center for Biomolecular Therapeutics (CBT), Department of Biochemistry and Molecular Biology, University of Maryland School of Medicine, Baltimore, MD 21201, USA; 2 Department of Physiology and Cellular Biophysics, Columbia University, New York, NY 10032, USA; 3 Biophysics Graduate Program, University of Maryland, College Park, MD 20742, USA; 4 Fischell Department of Bioengineering, University of Maryland, College Park, MD 20742, USA; 5 Department of Physics, Arizona State University, Tempe, AZ 85287, USA; 6 The Institute of Bioscience and Biotechnology Research (IBBR), Rockville, MD 20850, USA

**Keywords:** allostery, Ca^2+^-signaling, S100 proteins, calmodulin, binding and functional folding

## Abstract

EF-hand Ca^2+^-binding proteins (CBPs), such as S100 proteins (S100s) and calmodulin (CaM), are signaling proteins that undergo conformational changes upon increasing intracellular Ca^2+^. Upon binding Ca^2+^, S100 proteins and CaM interact with protein targets and induce important biological responses. The Ca^2+^-binding affinity of CaM and most S100s in the absence of target is weak (^Ca^K_D_ > 1 μM). However, upon effector protein binding, the Ca^2+^ affinity of these proteins increases via heterotropic allostery (^Ca^K_D_ < 1 μM). Because of the high number and micromolar concentrations of EF-hand CBPs in a cell, at any given time, allostery is required physiologically, allowing for (i) proper Ca^2+^ homeostasis and (ii) strict maintenance of Ca^2+^-signaling within a narrow dynamic range of free Ca^2+^ ion concentrations, [Ca^2+^]^free^. In this review, mechanisms of allostery are coalesced into an empirical “binding and functional folding (BFF)” physiological framework. At the molecular level, folding (F), binding and folding (BF), and BFF events include all atoms in the biomolecular complex under study. The BFF framework is introduced with two straightforward BFF types for proteins (type 1, concerted; type 2, stepwise) and considers how homologous and nonhomologous amino acid residues of CBPs and their effector protein(s) evolved to provide allosteric tightening of Ca^2+^ and simultaneously determine how specific and relatively promiscuous CBP-target complexes form as both are needed for proper cellular function.

## Overview

**Figure 1.**
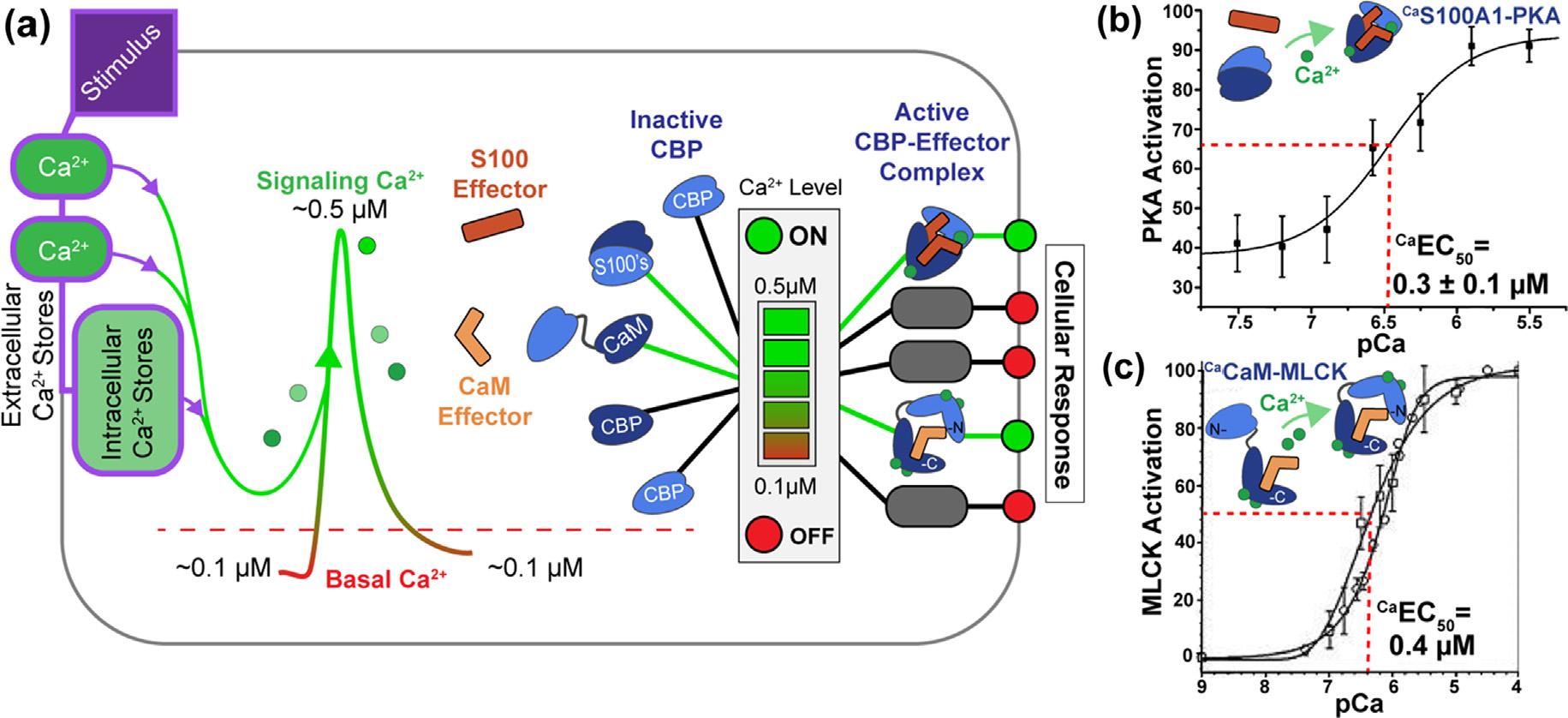
Schematic of Ca^2+^ binding proteins (CBPs) involved in intracellular Ca^2+^-signaling. (A) At [Ca^2+^]^free^ of ~0.1 μM, termed a resting concentration (red dashed line), CBPs, such as S100 proteins and CaM (light blue or navy-blue shapes), do not activate effector proteins (orange, red shapes). As [Ca^2+^]^free^ approaches or exceeds ~0.5 μM, active CBP-effector complexes form and provide a cellular response (green dot). (**B, C**) Illustrated are data showing activation of enzymes such as protein kinase A (PKA) by ^Ca^S100A1 (panel **B**) and myosin light chain kinase (MLCK) by ^Ca^CaM (panel **C**), respectively.^5–6^ More than 700 CBPs involved in Ca^2+^-signaling can be expressed in mammals as represented by light blue/navy shapes labeled CBP.

A major mechanism for transducing Ca^2+^ currents into biological function involves a family of Ca^2+^-binding proteins (CBPs) having a helix-loop-helix EF-hand structural motif. In many cases, CBPs bind Ca^2+^, undergo large conformational change (s), and interact with “effectors” or “protein targets” to elicit biological responses (Figure 1). CBP-effector complexes in turn activate enzymes, regulate ion channels, and deliver many biochemical and/or cellular signals. However, paradoxically, there are more than seven hundred EF-hand Ca^2+^-binding proteins in mammals alone, and they must be poised in any given cell type for rapid deployment upon an intracellular rise in free Ca^2+^ to generate specific biological function(s). Moreover, CBPs can reside in the cytoplasm at micromolar concentrations and are often very stable. Thus, with such an abundance of CBPs, at any given time, one might then ask, how is it possible that [Ca^2+^]^free^ is not depleted to levels lower than 100 nM, as is required for maintaining Ca^2+^ homeostasis? Secondly, how do functionally important CBPs compete for limited [Ca^2+^]^free^ when high levels of other CBPs are present? The answer to both questions is that CBPs bind Ca^2+^ weakly on their own (^Ca^K_D_ > 1000 nM) but are tightened allosterically (^Ca^K_D_ < 1000 nM) upon CBP-target protein complex formation (Figure 1).

**Figure 2.**
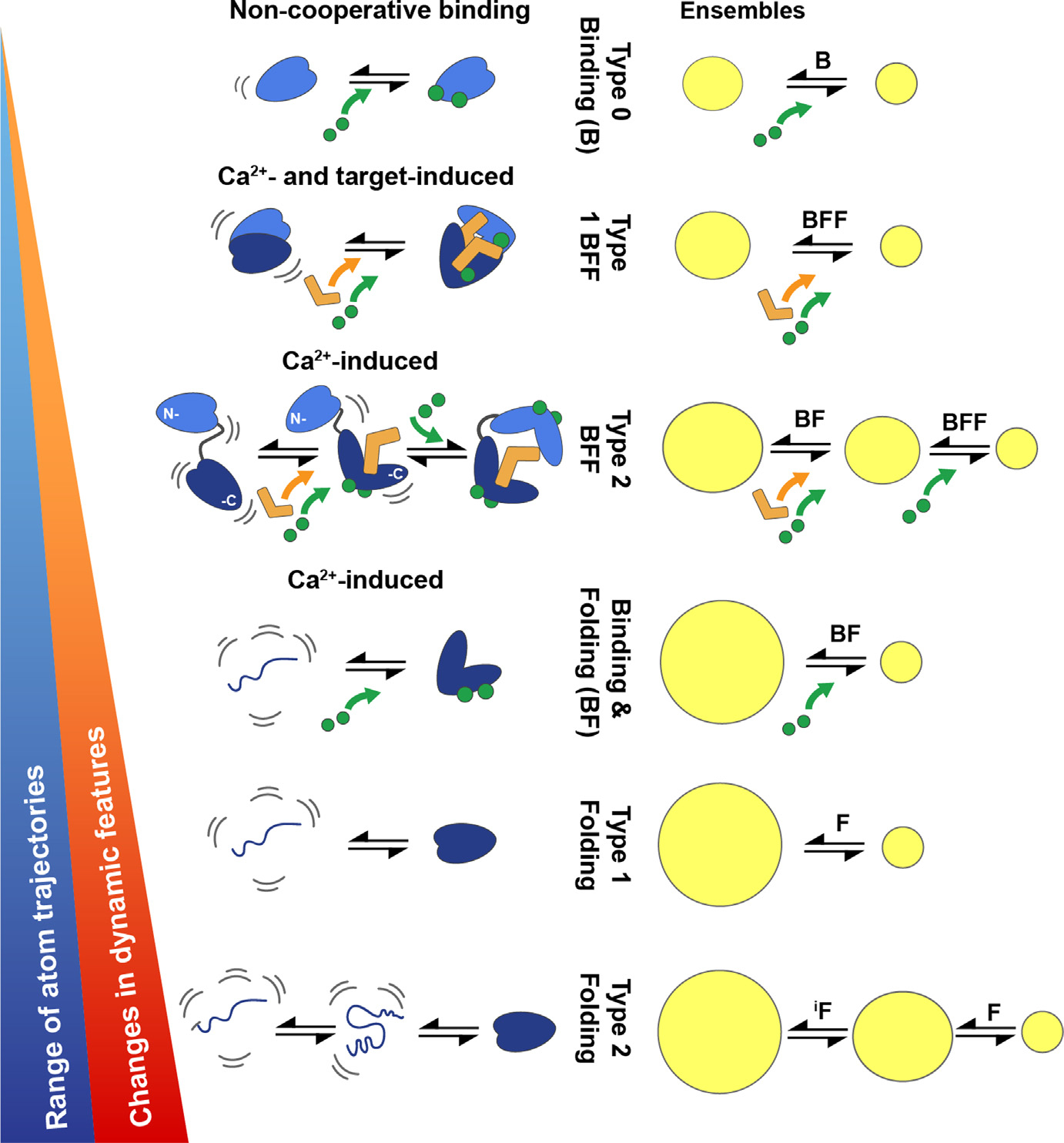
Cartoon illustrating the spread of atomic trajectories and the changes in the protein dynamics for all amino acid residues during functionally relevant binding and/or folding events. The events are categorized in a hierarchical manner from top to bottom, with those having the smallest change(s) in conformation, ensemble size, and in global and/or local dynamic character at the top. The events are labeled as pertaining to binding (B), folding (F),^4,16–18^ binding & folding (BF)^3,15^ and “Binding and Functional Folding” events (BFFs)^7–8^ for discussion purposes, but these events are not to be considered a complete list. For example, not shown in this figure are interactions involving chaperones during folding, which would represent another “Type” of folding (i.e., Type 3 Folding),^17–18^ and certainly there are also other types of BFs and BFFs in nature as well.

Two of many EF-hand Ca^2+^-binding protein subfamilies will be considered within a framework termed “binding and functional folding (BFF), the S100 and the CaM/CaM-like proteins^7–11^(Figure 2). The mechanism for a type 1 BFF is defined as being concerted and is represented by members of the S100 protein family, most of which increase their Ca^2+^-binding affinity allosterically upon CBP-target complex formation. A type 1 BFF mechanism ensures that a complex forms appreciably to “turn on” a proper cellular function(s) only when sufficient levels of the CBP, the effector protein target, and “activating levels” of free Ca^2+^ approaching ~500 nM are expressed or delivered into the cytoplasm, respectively (Figures 1(B) & 2). On the other hand, a type 2 BFF occurs via a stepwise mechanism, as illustrated for CaM.^12–14^ As with a type 1 BFF, timeframes relevant to cellular outputs are used to limit the number of possible states to only those that deliver or are on a pathway to deliver important cellular function(s). For example, the first step of a type 2 BFF involves a CaM binding and folding event (BF) at resting Ca^2+^ levels (~100 nM) to give a “functionally inactive” but stable CaM-effector complex (i.e., an intermediate),^3,15^ which is required and “on pathway” for the BFF event inside a cell. The second step represents the BFF step and produces the “functionally active CBP-target complex” as increasing intracellular Ca^2+^ levels approach ~500 nM (Figures 1(C) & 2).

## Structure-based models of Allostery: MWC and KNF

Many proteins, including calcium-binding proteins (CBPs) exhibit allostery (Figure 1), as reviewed extensively.^1,2,19–29^ The earliest models of allostery typically relied on how static protein structures could be correlated, as thermodynamic states, to cooperative binding curves of specific ligands, such as oxygen binding to hemoglobin tetramers.^30–31^ Two fundamentally distinct mechanisms arose including one from Monod, Wynam and Changeux, termed the MWC “symmetric” model, which invokes only quaternary structural changes among states to characterize allostery^32^. The second originated from Pauling and was extended by Koshland, Nemethy and Filmer, and is referred to as the KNF or Pauling-KNF model. The Pauling-KNF model provides allostery via sequential binding to distant sites of the hemoglobin tetramer with tertiary conformational changes in each subunit occurring at each binding event, which in turn, increases the affinity of molecular oxygen to the next subunit in the sequence of binding events^22,31,33^. Unlike the MWC model, the Pauling-KNF model offers mixed protomer states and affords broader allosteric properties. More complex mechanisms for allostery are also described using combination(s) of states from MWC- and KNF-like models.^22^

As X-ray crystallography structures of the relaxed (R) and tense (T) states for hemoglobin were refined, changes in the quaternary structures could be observed at atomic resolution that helped to explain its allosteric binding to molecules of oxygen.^34–39^ For example, salt bridges at the subunit interfaces were identified by Perutz and colleagues that showed how oxygen binding to the heme in the “T quaternary state” rearranged the position of a nearby helix, broke salt bridges at subunit interfaces, caused proton release, and destabilized the structure of the tetramer, thereby biasing the equilibrium toward the “R quaternary state”. In Perutz’s elegant structural description of allostery, the pH dependence of oxygen binding to hemoglobin, termed the Bohr effect, could also be described at the atomic level.^23,40–41^

As with studies of hemoglobin, allosteric Ca^2+^ binding to proteins in the S100 and CaM families has been well studied including with various adaptations of MWC/KNF-like models.^22,42–51^ Likewise, a deeper understanding of the energetics involving these biomolecular complexes were used to delineate thermodynamic energy contributions to Ca^2+^-regulated CBP-target complex formation via calorimetry techniques^52–58^, both for CaM^59–64^ and the S100 family of CBPs^7–8,43,65–66^. Thus, a solid foundation of biophysical data is in place for these and many other CBPs as is needed for their next step characterization at atomic resolution, including by considering dynamic ensembles and free energy landscapes.^2,21,29,59–60^

It is also an ideal time for using data from many techniques to examine allostery including biophysical, structural biology, and computational tools. For example, innovative and combined use of single particle cryoEM, X-ray, NMR, and other biophysical, biochemical, and advanced computational approaches has allowed for pushing the boundary of what complexes can be examined and now can more routinely include studies of large membrane-bound protein complexes^67–69^. Furthermore, interpretation of these data is highly reminiscent of approaches used to address the protein folding problem, so a next logical step here includes incorporating NMR and other experimental data together with applied mathematical modeling. This is particularly timely when considering recent successes with deep-learning (DL) and artificial intelligence (AI) methods for predicting protein folds.^70–73^

## Ensemble-Based Models of Allostery

Over recent years, major advances in allosteric modeling were achieved via “lessons learned” from the field of protein folding. As experimental approaches were improved and allostery moved towards treating single states as “dynamic ensembles” and transitions among states within funneled “energy landscape” descriptions. Although, as with protein-folding, what is often termed “Levinthal’s Paradox” needs to be considered. In essence, this paradox predicted very long protein-folding timeframes for searching all the possible degrees of freedom available to an unfolded sequence. In the folding field, the problem was addressed relatively easily via providing bias against locally unfavorable interactions even on the order of a few kT together with encoding sequence stabilization into the final lowest free energy structures as “local interactions”.^4,74^ It is these local effects together with “nucleation events” that lead to “funneled energy landscapes” and rapid folding times.^75–76^ Similar arguments can be made for the formation of biomolecular complexes particularly if relatively compact biomolecular interactions result from lesser compact dynamic ensembles, as with BF events (Figure 2). However, whether this holds true for all biomolecular BFF interactions remains a question. Also challenging are questions pertaining to distinguishing subtle changes in structure, dynamics, and their interconversions for predicting functionally relevant biomolecular complexes, which is a goal intensely under pursuit.^77–79^ Nonetheless, moving forward towards achieving such milestones will at a minimum start to populate data into “training sets” that may have some utility via AI/DL prediction algorithms as well as lead to other innovative approaches for distinguishing the energetic and structural details of complex biomolecular interactions, including those that are highly specific and/or allosteric in nature.

Despite such challenges, models of allostery via dynamic ensembles have improved as described by Hilser et al., Nussinov et al., and several others^2,74,80,21,81–82^. This includes parsing dynamic ensembles of protein conformations into energetically distinct states with varying dynamic features^2^. Such approaches are termed “conformational selection via population shift” or “ensemble allosteric” based modeling.^19,21,81,83^ Thus, with a range of kinetically competent conformational states available in solution, the association of ligands (i.e., ^ligand^k_on_), which can be fast or slow, can be sampled and effectively used to alter the free energy of states within various energy landscapes. Modeling allostery in this manner can be extended to consider effects of post-translational modifications and/or single/multiple amino acid changes, which may have arisen via evolution and/or from genetically derived diseases. It is this “search of states” as well as their “kinetic interconversion,” that will impact our ability to understand a larger variety of biomolecular complexes, in a more generalized manner as outlined in a “unified view of allostery” or by “allosteric networks”, respectively.^84–85^

Ensemble-based models and their corresponding energy landscapes can now supported experimentally as is needed for parameterizing various thermodynamic measurements, including from water, as well as time-dependent mechanisms,^86^ including binding kinetics (k_on_, k_off_) coupled to conformational change(s), and importantly the internal dynamic properties of biopolymers including those of proteins and protein complexes. Of particular importance is the application of NMR, which can provide atomic level information pertaining to the thermodynamic, structural, and dynamic properties of biomolecules, over more than 12 orders of magnitude in time (ps to > sec). Such data can be improved further by properly mimicking cellular conditions such as viscosity and other physiochemical properties including those relevant to the biology of biomolecules in normal and disease states.^87–88^ Importantly, NMR approaches are well suited for distinguishing fast-timescale motions (ps-ns) from conformational exchanging states (R_ex_occurring on slower time-scale motions (μs – ms;^19,80,89–96^. Although NMR data is most difficult to acquire in the μs timescale regime, binding and kinetic information is useful to help guide sparser amounts of NMR data on this biologically important timescale. Likewise, NMR provides a foundation for collecting data regarding the location(s) and dynamic properties for specific amino acid residues within a protein and to guide theoretical modeling of kinetic, thermodynamic, and other energetic properties.^92,97–104^

In addition to high-resolution structural data, novel X-ray crystallography and cryoEM methods are in place that can highly impact the characterization of allosteric biomolecular complexes. These include X-ray methods used to identify minor states in electron density^29,105–107^. Furthermore, single particle cryoEM, studies of larger dynamic complexes, including membrane proteins are now being examined readily^108–110^. With all these methods in place, ensemble-based descriptions of allostery are becoming even better equipped to consider conformational heterogeneity, including small or not even detectable conformational changes, as well as sequence-specific contributions to complex formation^2^ including those for large an biologically important membrane proteins^111–113^. This includes receptors and transporters allosterically regulated by CBPs such as CaM and the S100 protein family.^64,114–116^

This paradigm shift in the description of allostery as an ensemble of exchanging states is able to consider faster timescale motions by taking into account the thermodynamic effects of water^117–119^ and/or conformational entropy of the biomolecular complex within various energy landscape description(s).^2,60,120–121^ For example, the emergence of models such as “dynamic allostery,” as proposed by Cooper and Dryden, considered that entropy was involved as reflected by various protein fluctuations on multiple timescales and that enabled allosteric communication between distant sites even in the absence of defined/observable conformational changes. The consideration of conformational entropy was used to invoke mechanisms whereby ligand could induce changes in the internal dynamics of a protein among a number of thermodynamic states^122^. Pioneering studies by Wand and colleagues followed and identified dynamically defined methyl sidechains using NMR data that were classified based on varying trimodal distributions of rotamer states of methyl groups.^123^ They refer to these L-S squared generalized order parameters along the methyl symmetry axis as ${\text{O}}_{\text{axis}}^{2}$ versus with the model-free nomenclature, ${\text{S}}_{\text{axis}}^{2}$,^60^ and the three modes of dynamics are parsed as what are termed J-class, ω-class, or aα-class parameters for qualitatively identifying states and for measuring contributions to entropy during complex formation. In the J-class, the Lipari-Szabo squared generalized order parameters for the symmetry axis of the methyl groups have a distribution of values at ~0.35 and represents extensive rotamer interconversion on fast-timescales (<ns) while those methyl groups having a distribution of order parameters of ~0.8 have little if any fast timescale rotamer interconversion on this timescale. Those methyl groups in the α-class have squared order parameters of ~0.6 and are suggested to involve large-amplitude motions within one rotamer state, but they may also have occasional rotamer jumps^60,123^. In summary, such data allows for defining states from a global picture of the rotamer dynamics, and their changes upon complex formation can be used to obtain entropic contributions to biomolecular complex formation in a highly rigorous manner^13,59–60,119–121,124–127^. CaM served as an early model for calibrating this “entropy meter” method with NMR and calorimetry^60,121^, and thus a very useful background of knowledge regarding this CBP and its complexes is already in place for use in the BFF framework for treating allostery.^13,60,124–128^

While many binding models postulate that allostery is propagated at a residue-specific level, the identification of a subset or network of residues that dominate the allosteric mechanism within a folded protein is often a goal.^84^ In such treatments, critical residues are defined and are correlated based on dynamical information to provide an allosteric pathway(s) linking the two distant sites. However, unlike these models, which focus on “critical residues” along a pathway between sites, the BF and BFF frameworks consider instead a more global consideration of ensembles, which are analogous in some ways to a “folding landscape”, but they include all residues within both components of the biomolecular complex. Such an “all sequence” treatment for modeling allostery has the added benefit of deriving how specificity is achieved for biomolecular complexes. Thus, a “folding-like” framework for treating all atoms within a protein ensemble and energy landscape is preferable, so that understanding details of complex formation, its lifetime for functioning, and its dissociation can be understood at the molecular level in a global and “full-sequence” manner.

## Genomic considerations for understanding functional protein complexes

It is essential that evolution is not ignored when considering protein folding and biomolecular interactions (Figure 1). It is now understood that there are approximately 10^6^ protein sequences, at most, and their pairwise and/or higher order interaction(s) encode for approximately 10^3^ to 10^4^ functionally stable protein folds^129–131^. As such, evolutionary pressure to conserve physically attainable folds via amino acid sequence is recognized as the “fold over function” postulate of evolution, but such an observation is not sufficient for understanding how biological function arises from these protein scaffolds and the complexes that they form.^132^ With an understanding that function guides how sequences evolve, “full sequence approaches” are in demand to address this need, which include homologous and nonhomologous regions. Particularly when considered together with biophysical, structural, dynamic, and computational approaches, existing and innovative new genomic approaches can significantly impact how we derive molecular mechanisms for how biomolecular complexes achieve function including via any allostery associated with these complexes.

A distinguishing difference between CaM and the S100 family from a genomics perspective is that CaM is one of the earliest genes known in time, with many of the earlier detected genes also being CBPs, other than those in the S100 family. Furthermore, sequence consistent with CaM-target complexes, such as with a cyclic nucleotide phosphodiesterase, are also found to be unvaried as far back as in the Metazoan and in Eucaryota eras^133^. Whereas S100 sequence did not show up in numbers until vertebrates.^134–135^ While the functional relevance of this information is not completely clear, it is postulated that the earlier CBPs, such as CaM, troponin C (TnC), and others provided multiple functional purposes as second messengers in cellular Ca^2+^-signaling; whereas, those in the S100 family evolved for more specific functions as may be required of more highly complicated organisms.^134–135^ In fact, one potential role for S100s in humans, in general, is that they may contribute to regulating CaM-mediated activities via direct competition, as was observed for ryanodine receptors of skeletal muscle (RyR1) and heart (RyR2).^62,136–140^

It must be cautioned, however, that care must be taken since coevolution of structure and associated functions is often obscured by artifactual signals such as genetic drift, which is caused by inter-clade and intra-clade sequence comparisons, which demonstrates that coevolution can be measured on multiple phylogenetic timescales within a single protein. This phenomenon predicts functional coevolution to be more difficult to detect and often opaqued by structural-contact predictions. While beyond the scope of this review, newly developed genomic methods, calculate nested coevolution (NC) to separate inter-clade and intra-clade sequence comparisons via novel energy landscapes that minimize noise and can detect allosteric coevolved mutations as well as other functional outputs.^141^

Likewise, biochemical experimental studies can be employed via use of systematic pairwise, and higher order mutagenesis studies to delineate whether pairs of amino acid residues are interacting cooperatively, are restrained in an anticooperative manner, or non-interacting in various binding modes. Such methods are particularly impactful to identify networks of residues involved in transition-state stabilization of enzymes since activation free energy barriers can be as high as 16 kcal/mol, in the case of phosphodiester hydrolysis^142–144^. Thus, mutant cycle studies, genomic, and evolutionary considerations are impactful for predicting and detecting cooperative processes and allostery, including most notably within the active sites of enzymes.^145–146^

## A model system for understanding biomolecular interactions and allostery within the BFF physiological framework

**Figure 3.**
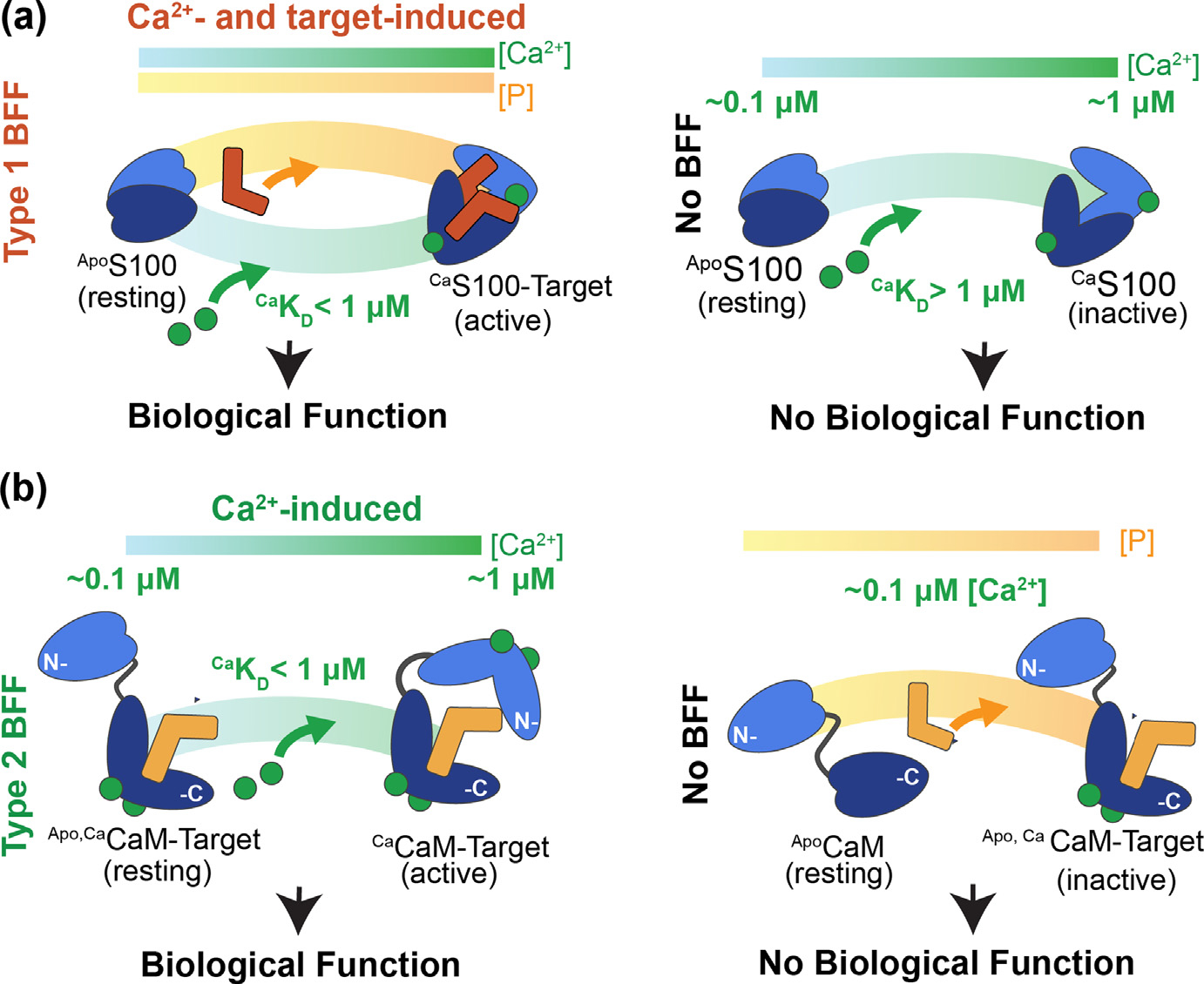
Two types of Binding and Functional Folding (BFF). (A) A concerted Type 1 BFF is illustrated for CBPs in the S100 protein family (blue; left panel). If the effector protein is not available (right panel), then the S100 protein does not affect biology, which includes not appreciably sequestering any free Ca^2+^ (^Ca^K_D_ > 1 μM). These CBPs do not appreciably interact with target at resting [Ca^2+^]^free^, but when Ca^2+^ levels are elevated (~0.5 μM) and an effector protein is present, then biological function occurs (see also Figure 1B)^5^. (B) A stepwise Type 2 BFF is illustrated for CaM.^12,14^ In step 1, prior to a Ca^2+^-signaling event, at resting Ca^2+^ concentrations (0.1 μM), the Ca^2+^-bound C-lobe of CaM (dark blue) binds to its target (light orange) in as part of a BF interaction (Figure 2) to give but remains in an inactive form (^Apo, Ca^CaM-Target; right panel). In step 2, upon a rise in intracellular Ca^2+^ during signaling (~0.5 μM, arrow; left panel) a Type 2 BFF is triggered, as found for STRA6 BP2^64^, MLCK^6,12^ and other CaM targets.^161–164^

There are several reasons that biomolecular complexes involving CBPs are well suited to be used as models for studying biomolecular interactions and allostery. First, there is an abundance of biophysical, thermodynamic, and atomic level structural data available for these complexes, including via X-ray crystallography, NMR, and by cryoEM. Secondly, CBPs are relatively small, and amenable to sophisticated computational approaches, via all atom approaches, including those that can now approach the μs – ms timescales^147^. CBP-target complexes have relatively straightforward allosteric mechanisms of action, including those of the S100 family (type 1; concerted) and those involving CaM (Type 2; stepwise)^8,60,148–160^ (Figure 2). There are examples of complexes that are highly specific and those that are not, allowing for this important component of biology (i.e., specificity) to be explored. Finally, and most importantly, the biological relevance of CBP-target complexes is far reaching as they are critically important in all cell-types throughout nature, including towards the evolution of humans. Two of the most straightforward types of allosterically driven CBP-target complexes will be considered here to illustrate the BFF framework (Figures 2, 3: (1) a concerted BFF (type 1) and a stepwise BFF (type 2). The type 2 BFF first employs a binding & folding event (BF) to form an inactive, but stable intermediate inside the cell (BF; step 1); however, the intermediate state (I) is functionally relevant since it is on the pathway towards a functional state, in the second BFF step (BFF; step 2).

### Type 1 BFFs (Concerted): Functional biomolecular complexes involving S100 proteins.

A type 1 BFF event is concerted as represented by Ca^2+^-dependent S100-target complexes, which do not form unless sufficient concentrations of the S100 protein, target protein, and elevated free Ca^2+^ levels (>500 nM) are present simultaneously within the cytoplasm of a cell (Figure 3). Moreover, in the absence of target, the Ca^2+^-binding affinity for most S100 proteins is relatively low (K_D_ > 1000 nM), and importantly, they do not sequester significant quantities of free Ca^2+^ from the intracellular pool unless their functionally relevant molecular target(s) is/are available. This allosteric property is significant physiologically, as there are approximately thirty EF-hand Ca^2+^-binding proteins in the S100 protein family in mammals, many of which are present at micromolar concentrations within the cell, and they are at even higher levels in many disease states.^8,151^

S100 proteins were named since the earliest family members were found to be 100 % soluble in ammonium sulfate. These structurally homologous proteins typically form homodimers via an X-type four-helix bundle fold in solution, at concentrations less than pM,^165^ with the lone exception being calbindin D_9_k, which is monomeric in solution even at mM concentrations.^156^ However, subunits of S100s do not typically bind Ca^2+^ cooperatively nor does S100 dimerization contribute to allostery target binding or any allostery regarding increasing Ca^2+^-binding affinity^8^. The target binds each S100 subunit typically in a 1:1 stoichiometry and does so in a noncooperative manner, and the S100-target complex is almost always fully symmetric, which can be easily validated by NMR.^8^ In contrast to CaM, S100s are expressed exclusively in vertebrates and often exhibit a high level of specificity for their protein targets, thus allowing for the regulation of many biological pathways in a cell-specific manner.^151^ While CaM can bind to some targets in the absence of Ca^2+^, those in the S100 protein family rarely do so, but more typically undergo Ca^2+^-dependent conformational change as a requirement for binding target proteins.^5^ S100 proteins also represent a useful and relatively simple model for examining allostery since each S100 proteins contains only a single physiologically relevant canonical EF-hand Ca^2+^-binding domain per subunit that is in its C-terminus and involving the EF-hand helix-loop-helix Ca^2+^-binding domain involving helices 3 and 4. In summary, the allostery within this family of proteins is relatively straightforward, and thus a very good model system to consider first within the BFF framework outlined here.

In the Ca^2+^-free form, S100 proteins are in a “closed” conformational state with the target binding site blocked. This family of proteins contains EF1, an N-terminal “pseudo” or “S100” EF-hand (14-residue), which exhibits lower Ca^2+^ affinity and EF2, a C-terminal “canonical” EF-hand, which exhibits higher Ca^2+^ affinity.^166–167^ Upon binding Ca^2+^, helix 3 of S100B reorients ~90 degrees relative to helix 4 of EF2 and exposes a hydrophobic pocket that is important S100-target binding (PPIs; Figure 5). In the absence of a molecular target, S100 proteins typically have a low affinity for Ca^2+^ (K_D_ ≫ 1000 nM) allowing them to exist at micromolar concentrations inside the cell without depleting free Ca^2+^ levels and “short-circuiting” Ca^2+^ oscillations (Table 1). Thus, very low levels of ^Ca^S100 proteins are “poised and ready” to respond to rises in free Ca^2+^ associated with cell-signaling events, but ^Ca^S100B-target complex can only appreciably form when their specific effector protein is expressed and available at sufficient levels inside the cell. CBP-target complex is required for an S100 protein to sequester free Ca^2+^ inside a cell since it is only when the S100 target is bound that the Ca^2+^ affinity for its single EF-hand (EF2) is high enough to compete for intracellular free [Ca^2+^]. Representative S100-target complexes are listed for full-length S100-target complexes involving S100A1, S100B, and S100A4 (Table 1).

As with other EF-hand calcium-binding proteins, most S100 proteins have an increased affinity for Ca^2+^ in the presence of a molecular target.^136,169–171^ Importantly, when interactions with larger peptides and/or full-length targets are examined, Ca^2+^ binding affinities are tightened by as much as two orders of magnitude as needed to be functional during an intracellular Ca^2+^ signaling event in the 100 to 1000 nM range; whereas, S100 interactions with smaller domains or peptides do not always achieve a functionally relevant state (Table 1). Such observations alone, with peptide-binding experiments, are indicative that sequences in the target protein beyond those involving the major PPI binding site are needed to fully engage and provide a functionally relevant allosteric effect for members of this protein family. As specific examples, functionally relevant S100-target complexes were observed involving the canonical EF-hand (EF2) of ^Ca^S100A4-p37 and ^Ca^S100A4 nonmuscle myosin IIA, which bind Ca^2+^ at nM concentrations ranging from 200–300 nM.^48,66,174,177^ A second example is with ^Ca^S100A1, which is an S100 protein shown to activate skeletal muscle protein kinase A (PKA), even in the absence of cAMP, and at physiologically relevant Ca^2+^ concentrations (~300 nM)^5^, and there are many more.^8,151^ In the case of ^Ca^S100A1, specificity of ^Ca^S100A1 for PKA RIIβ (versus ^Ca^CaM, ^Ca^S100B) and allosteric tightening of Ca^2+^ was fully apparent in this highly specific S100-target protein interactions,^5^ so studies of this ^Ca^S100A1-RIIβ complex (Figure 1(B)) represents an opportunity to study allostery and specificity of complex formation simultaneously.

**Figure 4.**
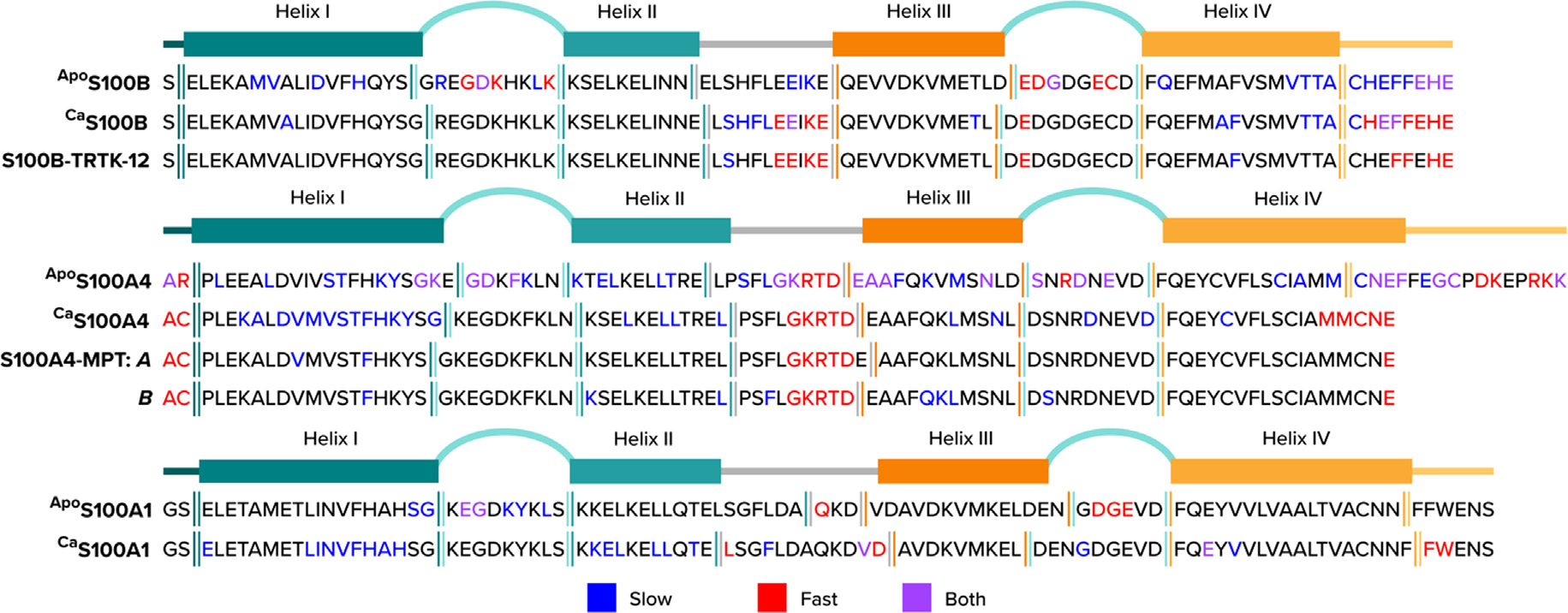
Dynamic properties of S100s in the absence and presence of Ca^2+^ and/or target peptide. Secondary structures of S100B, S100A4, and S100A1 are shown along with the corresponding sequences in the absence and presence of Ca^2+^. In red are residues that exhibit fast time scale motion (ps-ns) determined from either S^2^ (for S100A4 and S100A1) or NOE measurements < 0.75 (S100B). Residues with slow timescale motion (μs– ms) are shown in blue and were identified by relaxation dispersion NMR measurements (R_ex_). ^Apo^S100A4 was derived from mouse and Ca S100A4 in the absence and presence of nonmuscle myosin IIA derived peptide (MPT) was derived from human and is missing 13 residues from the C-terminus^48,181^. MPT binds S100A4 asymmetrically, so the two monomers (A, B) were evaluated separately.^48^

**Figure 5.**
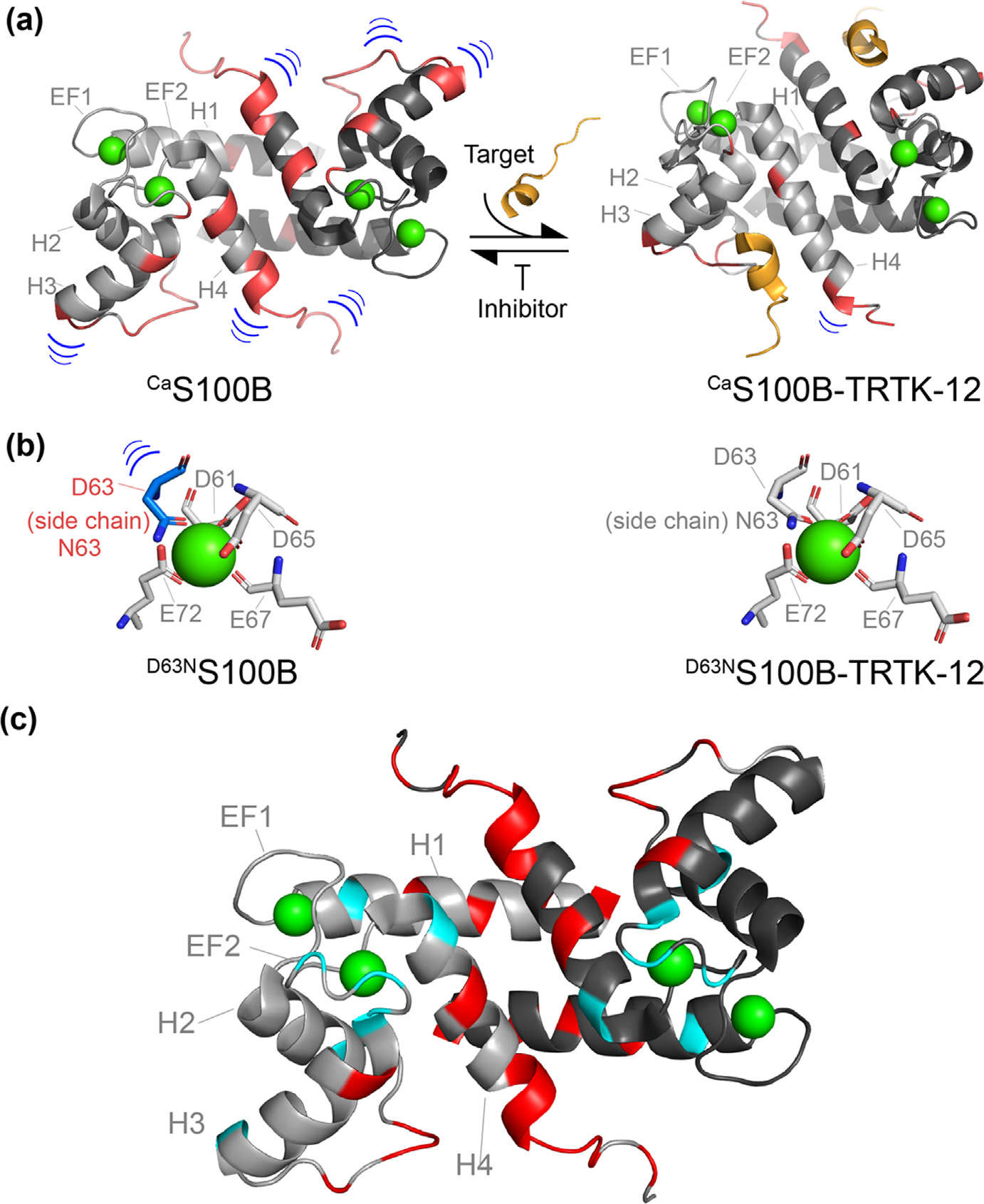
Changes in dynamic properties of ^Ca^S100B upon binding TRTK-12. (A) Residues exhibiting fast, and slow timescale motion are highlighted in red on ribbon diagrams for ^Ca^S100B, and ^Ca^S100B-TRTK-12 (grey). A target peptide such as TRTK-12 (orange) reduces the dynamic properties of S100B throughout the entire protein sequence, including for residues within EF2, as shown by blue contours. (B) Ca^2+^-coordinating residues within EF2 are shown in a diagram where N63 of ^Ca^S100B^D63N^ exhibits slow timescale motion (blue), which decreases upon binding TRTK-12 and contributes to Ca^2+^-tightening. (C) A ribbon diagram of the NMR structure of ^Ca^S100B is illustrated with residues colored in red that show reduced motions on fast (ns-ps) or slow (μs-ms) timescales upon addition of a peptide derived from CapZ, termed TRTK-12. Similarly, side chain amide correlations for Asn or Gln, which show a decrease or loss of chemical exchange upon TRTK-12 addition are highlighted in cyan.

Insights regarding allosteric Ca^2+^-binding to S100 proteins were uncovered using NMR for several S100 proteins, including S100B, S100A1, S100A4, and S100A5.^7,178–179^ At first, this included studying motions ranging from ps to ms timescales in the absence and presence of Ca^2+^ in both EF-hand Ca^2+^ binding sites for S100A1^180^, S100B^97^, S100A4,^181^ and S100A5 (Figure 4).^20^ In each case, Ca^2+^ binding to the S100 protein in the absence of a target, most often decreased and/or eliminated dynamic features in the EF-hand Ca^2+^ binding loops for ^Ca^S100A1, ^Ca^S100B, ^Ca^S100A5, ^Ca^S100A4, and for ^Ca^calbindin D_9_k on a range of timescales (Figure 5).^7,20,48,182–184^ In contrast, dynamic properties in the “hinge (loop 2) and C-terminal region were not quenched upon Ca^2+^ binding, and in some cases, including ^Ca^S100A1 and ^Ca^S100B, the addition of Ca^2+^ actually provided additional dynamic features upon binding Ca^2+^, on both fast and slow timescales.^7,20,182^ Moreover, Ca^2+^ binding to ^Apo^S100A1 and ^Apo^S100B enhanced slow timescale motion for several residues two loop regions (loop 2, 4) and in helices I, II and IV of ^Ca^S100A1 upon comparison. Furthermore, the most pronounced of these Ca^2+^-initiated motions were found mostly in nonhomologous regions of the S100 family including for residues loop 2, also termed the “hinge” region and the C-terminal loop (loop 4), which is also not highly conserved when sequences in S100A1 and S100B proteins are compared. These findings plus the fact that these loop regions were important for target protein binding provided hypotheses that such dynamic features in these loop regions could potentially be important for molecular recognition, and also provided some rationale for target binding specificity among this protein family inside a cell.^159,172,185–187^

To examine S100-target complexes in more detail, rigorous NMR relaxation studies were collected and compared for ^Ca^S100B and Ca S100A4 in the absence and presence of peptide targets. As it turns out, these studies provided information about molecular recognition for this complex, but they also provided novel insights for how target binding to an S100 protein could allosterically increases its Ca^2+^-binding affinity.^7,48^

**Figure 6.**
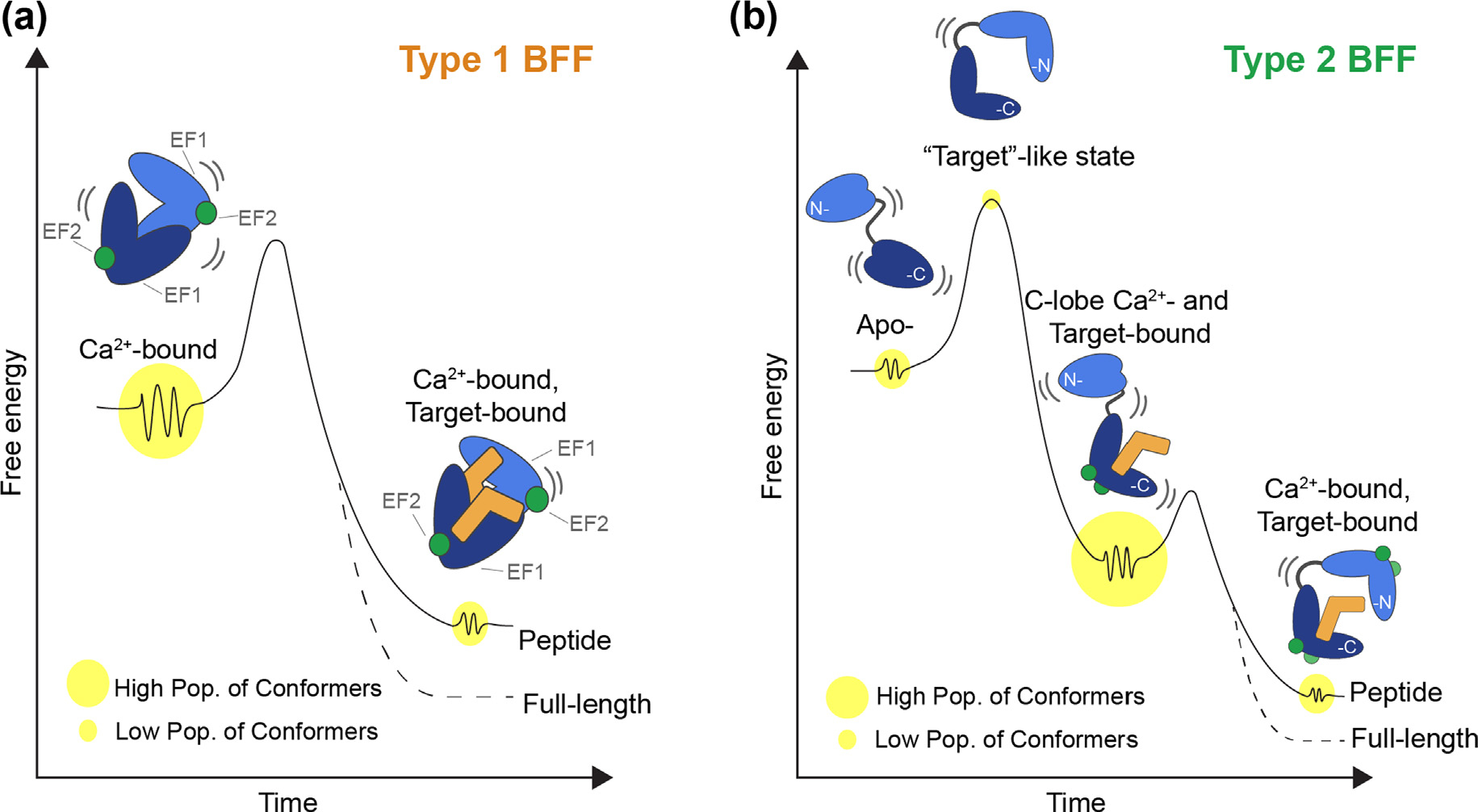
Free energy diagram for allosteric Ca^2+^/target binding to EF-hand Ca^2+^-binding proteins. (A) A free energy diagram for a concerted Type 1 BFF mechanism is illustrated that involve members of the S100 family. (B) A free energy diagram for a stepwise Type 2 BFF mechanism is illustrated that involve CBPs of the CaM or CaM-like families. As illustrated in both panels, free energy diagrams for Ca^2+^-dependent CBP-Effector interactions involving full-length effector complexes are likely tuned differently (dashed line) than those of peptides derived from full-length target/effector complexes (solid lines), so numerous controls must be completed when interpreting data for peptide complexes, as described previously.^64,114^

In the absence of a peptide from nonmuscle myosin IIA, termed the MPT peptide, many residues of helix I of S100A4 exhibited slow timescale conformational exchange (R_ex_), but this R_ex_ was reduced significantly upon MPT binding. Of particular interest were observations made for residues in helix I, which is not part of the target recognition site, but instead makes up part of the dimer interface. From these data, it was interpreted that reduced dynamic properties of helix I in the presence of peptide likely contributed to S100A4 dimer stabilization versus having any direct effects on Ca^2+^ binding. Nonetheless, changes in dynamic properties were found throughout the sequence of ^Ca^S100A4 rather than via residues originally hypothesized to link target-binding to Ca^2+^-binding as part of the allosteric effect. Likewise, many residues of ^Ca^S100B in helix I, the hinge region, helix IV, and the highly mobile C-terminal tail, show reduced slow timescale motion (less R_ex_) in the presence of a consensus S100 sequence, TRTK-12, versus in its absence (Figures 5, 6), again showing global changes throughout the entire sequence of the protein upon the addition of a peptide target.^7^

Importantly, to link these changes in dynamic features to residues within EF2 of an S100 protein, ^15^N sidechain motion in a Ca^2+^ coordinating residue was examined in the absence and presence of a target molecule.^7^ Specifically, for ^Ca^S100B, a D63N mutant (^Ca^S100B^D63N^) was studied since the ^15^N-atom on the sidechain of this asparagine residue could be readily detected and monitored via NMR. As a control, the D63N mutant was shown to coordinate Ca^2+^ in a manner like that of the wild type S100B via X-ray crystallography, and it was found indistinguishable Ca^2+^ binding properties when compared to the wild-type protein. Importantly, the sidechain motion of ^D63N^S100B was found to be mobile in the absence of the target peptide, but static upon binding to TRTK-12, as is consistent with it coordinating at the third position of the EF-hand upon binding of the protein target and thus at least partially demonstrating allosteric tightening observed in the kinetic and thermodynamic Ca^2+^-binding experiments^7,171^ (Figure 5).

In addition to changes in dynamics discovered for this coordinating residue, many other residues throughout the entire sequence of S100B, both in homologous and nonhomologous regions, showed a significant reduction in their dynamic properties (Figure 5). In summary, both ^Ca^S100A4 and ^Ca^S100B exhibited dynamic properties in the absence of target across multiple timescales, which were eliminated or changed in the presence of molecular target, including for ^Ca^S100B at a Ca^2+^-coordinating residue, upon TRTK-12 addition, as is consistent with allosteric tightening from changes on a more global scale. A second important dynamical feature is that TRTK-12 and MPT peptides both themselves underwent a disordered to ordered transition in which they both transitioned from a random coil to a helical structure, consistent with a binding and functional folding mechanism for allostery.^3,48,169^ As with S100s, a similar phenomenon as this was observed for several CaM target peptides, which gain helicity upon binding to ^Ca^CaM.^12,188–191^ In summary, structural, and dynamic changes occurred throughout both the CBP and the target peptides in a concerted manner, with some of these changes observed directly in the coordination sphere of the EF2 Ca^2+^-binding site, as is consistent with the allosteric tightening of Ca^2+^-binding.

It was important to distinguish that Ca^2+^-tightening observed upon target peptide binding was achieved by reducing dynamic properties of Ca^2+^-coordinating residues versus an alternative mechanism in which additional coordinating residues are added, as is found for some of the tightest of CBPs found in nature, such as parvalbumin.^192–193^ Specifically, an alternative explanation for the tightening of Ca^2+^ is that the overall coordination of Ca^2+^ was more optimal when the target was bound. Specifically, the scenario that a ligand to Ca^2+^ that is typically occupied by a water molecule in most EF-hand proteins could possibly be coordinated by the S100 itself. This possibility was ruled out for the ^Ca^S100B- and ^Ca^S100A4-complexes since in both cases the Ca^2+^ coordination was found to be identical, with the same water occupancy, whether the target peptide was bound.^7,48^

For the ^Ca^S100B-TRTK complex, additional information pertaining to the dynamic ensembles was examined, including the potential for the existence a pre-equilibrium between closed (^Apo^S100) and open (^Ca^S100) states of S100 proteins prior to Ca^2+^ binding, whereby target-binding shifted the equilibrium towards the Ca^2+^-bound state.^7^ However, in the absence of Ca^2+^, there was no evidence of S100B binding to TRTK-12 or for S100A4 binding to MPT^7,66^ even at mM concentrations nor was any pre-equilibrium detected via fluorescence measurement in the case of the S100B-TRTK12 complex, which were completed at concentrations as low a 10 nM (data not shown). It is for these reasons that only the ^Ca^S100 state is considered for the dynamic ensembles involving the BFF type 1 mechanism of action, and the ^Apo^S100 state is not.

Similarly, ^Ca^S100A4 did not exhibit slow motion at the Ca^2+^ coordinating residues versus ^Apo^S100A4, also implying that there is no chemical exchange between the open and closed states as part of its S100-target dynamic ensemble for this S100 in the absence of Ca^2+^. Furthermore, ^Apo^S100B did not exhibit R_ex_ for residues in EF2 or helix III, which would be expected and important for hydrophobic site exposure upon binding Ca^2+^, as would be predicted if a pre-equilibrium did indeed occur. Together, these studies indicate that a pre-equilibrium between open and closed states for these S100 proteins is unlikely to contribute to Ca^2+^ tightening within detection limits of the relaxation dispersion data set collected (<1%).

For another S100 protein, S100A11, deuterium-hydrogen exchange mass spectrometry data (HDX/MS) was collected and used to define another Type 1 BFF. In this case allostery originating from outside the target binding site involved a salt bridge between K32 and D57. In this example, Ca^2+^ binding site was found to destabilize interactions of adjacent residues, propagating a cascade of events resulting in the target binding-site to actually close.^194^ Whereas the binding of Ca^2+^ reduced fluctuations of residues involved in the cascade, causing the target binding site to stay open. As with S100A1 and S100A4, in the other direction, this group showed that peptide binding to ^Ca^S100A11 also was sufficient to stabilize a H-bonding network within the EF hands of S100A11, which correlated with increased Ca^2+^ affinities observed in the presence of the peptide target.^194^

There are highly practical reasons for understanding allosteric biomolecular interactions since it presents an opportunity to regulate them if such an interaction promotes a disease state. For example, basal expression of S100B is known to be neuroprotective^195^, but increased expression often correlates with disease states including multiple cancers such as malignant melanoma and colorectal cancer,^8^ as well as brain injury, neurodegenerative diseases and neuropathies like Parkinson’s disease, Alzheimer’s disease, mood disorders^196^, and schizophrenia.^197^ Similarly, S100A1 enhances muscle contraction in cardiac and skeletal muscle, but decreased S100A1 is associated with heart failure^170,198–199^. However, in other disease states, overexpression of S100A1 is associated with neuropathology of the brain^200^. The upregulation of S100A6, which is found in muscle, kidney, spleen, brain and the lungs^201^, is associated with several cancers including breast, pancreatic and colorectal cancer.^8,202^ S100A4, which is related to cell migration and cell proliferation, has been detected in breast, liver, and brain metastases^203–205^. Like S100B, increased S100A4 levels is linked to poor survival in cancer patients.^206–207^ Therefore, developing S100-specific inhibitors is of great interest to counteract these disease states, and thus understanding how to interrupt these allosteric biomolecular interactions represents an opportunity to address such diseases therapeutically.

For illustrative purposes, S100B is a marker for malignant melanoma (MM), and one potential mechanism of action for its causal effect on MM is that it binds to the cell cycle regulator, p53, blocking its tumor-suppressive function.^172,208–209^ Thus, developing S100 protein-specific inhibitors, including for S100B, is of great interest to treat a variety of diseases in which S100B protein levels are elevated. To this end, three persistent target protein-binding sites (termed Sites 1–3) within S100B were identified and inhibitor development to block these sites and restore p53 activity is ongoing^64,171,210–211^. In this case, the goal of the inhibitor design is to target S100B, but avoid other S100s such as S100A1, which are important for other important biological functions, such as cardiac and skeletal muscle function.^198^ Thus, a complete understanding of allosteric CBP-target interactions that are specific for one versus another S100 protein will significantly facilitate this goal and are ongoing to advance the engineering of S100-specific inhibitors for cancer as well as other disease states.

### Type 2 BFFs: Functional biomolecular complexes involving CaM and CaM-like proteins.

Ca^2+^dependent interactions of CaM with a large number of biological targets are reviewed,^161–164,212^ and prototypical allosteric complexes involving ^Ca^CaM such as the ^Ca^CaM-MLCK peptide,^51 Ca^CaM- STRA6 BP2^64^ and many others occur in a stepwise manner (Figure 2) and are termed here Type 2 BFFs.^12–14^ It is understood that side chains of ^Ca^CaM are some of the most dynamic of any protein yet measured, and not surprisingly ^Ca^CaM is highly promiscuous and has many protein targets with varying sequences. Surprisingly, it can also bind to peptide targets synthesized with D-amino acids.^128,213^ Whereas, the S100 family members often provide highly specific interactions, as most readily demonstrated when full length CPB-target complexes are examined in side-by-side comparison (Figure 1(B)).^5^

CaM is a small (16.8 kDa), ubiquitously expressed transducer of Ca^2+^ signals inside the cell^164,214–215^. Highly conserved in all vertebrates, CaM is encoded by three genes that are translated into three identical, redundant sequences each made up of 148 amino acids.^216^ The structure of CaM has two globular domains, known as the N-terminal domain (N-lobe) and C-terminal domain (C-lobe), each containing two EF-hand Ca^2+^ binding sites (named EF1–4). EF1 and EF2 of the N-lobe bind Ca^2+^ 6 to 7-fold weaker than EF3 and EF4 of the C-lobe (^Ca EF1/EF2^K_D_ = 13 μM; ^Ca EF3/EF4^K^D^ = 2 μM; Table 2)^51,217–218^. A flexible linker connects the two CaM domains that allows the CaM to assume a large array of different conformation states prior to binding to its target proteins.^161,217,219^

In the absence of Ca^2+^ and target, Ca^2+^-free CaM (^Apo^CaM or ^Mg^CaM) is described as having both the N- and C-lobes existing in a “closed” conformation, where hydrophobic residues of CaM form a hydrophobic core and the interhelical angles of EF1–4 are between ~130–140° (Table 3)^220^; however, little if any ^Apo^CaM exists inside a resting cell where Mg^2+^ concentrations can approach millimolar (mM) levels, so any Ca^2+^-independent interactions of CaM should be examined in the presence of mM concentrations of Mg^2+^ (^Mg^CaM).^64^ Furthermore, Ca^2+^/Mg^2+^ exchange withing the EF-hand domains represents another physiologically relevant activity that is important to consider for CBPs.^64,221–222^

It is established that Ca^2+^-independent CBP-target interactions occur involving CaM.^161–162,223^ Most notable are interactions involving target proteins containing IQ-motifs (IQxxxRGxxxR).^161^ Many of these CaM-IQ motif complexes involve hydrophobic interactions from the interior of the C-lobe of ^Apo^CaM or ^Mg^CaM and via hydrogen bonds to the CaM surface.^224–225^ A “semi-open” conformation represents an intermediate of the closed and open state (interhelical angles 110–120°), and it is reported for the ^Apo^CaM C-lobe in the presence of these IQ motif targets (Table 3)^226^; whereas, a “closed” conformation occurs typically in the N-lobe. Important for this discussion, the binding of IQ-motif-containing proteins such as neurogranin, in the absence of Ca^2+^, has little if any effect on Ca^2+^ binding, and in fact, for some cases involving IQ motif targets, decreases in Ca^2+^-binding affinity are observed upon target binding (Table 4).^223,227–230^ Thus, the allostery upon target binding in these systems is small, if at all, in comparison to most prototypical CaM-target complexes.

**Figure 7.**
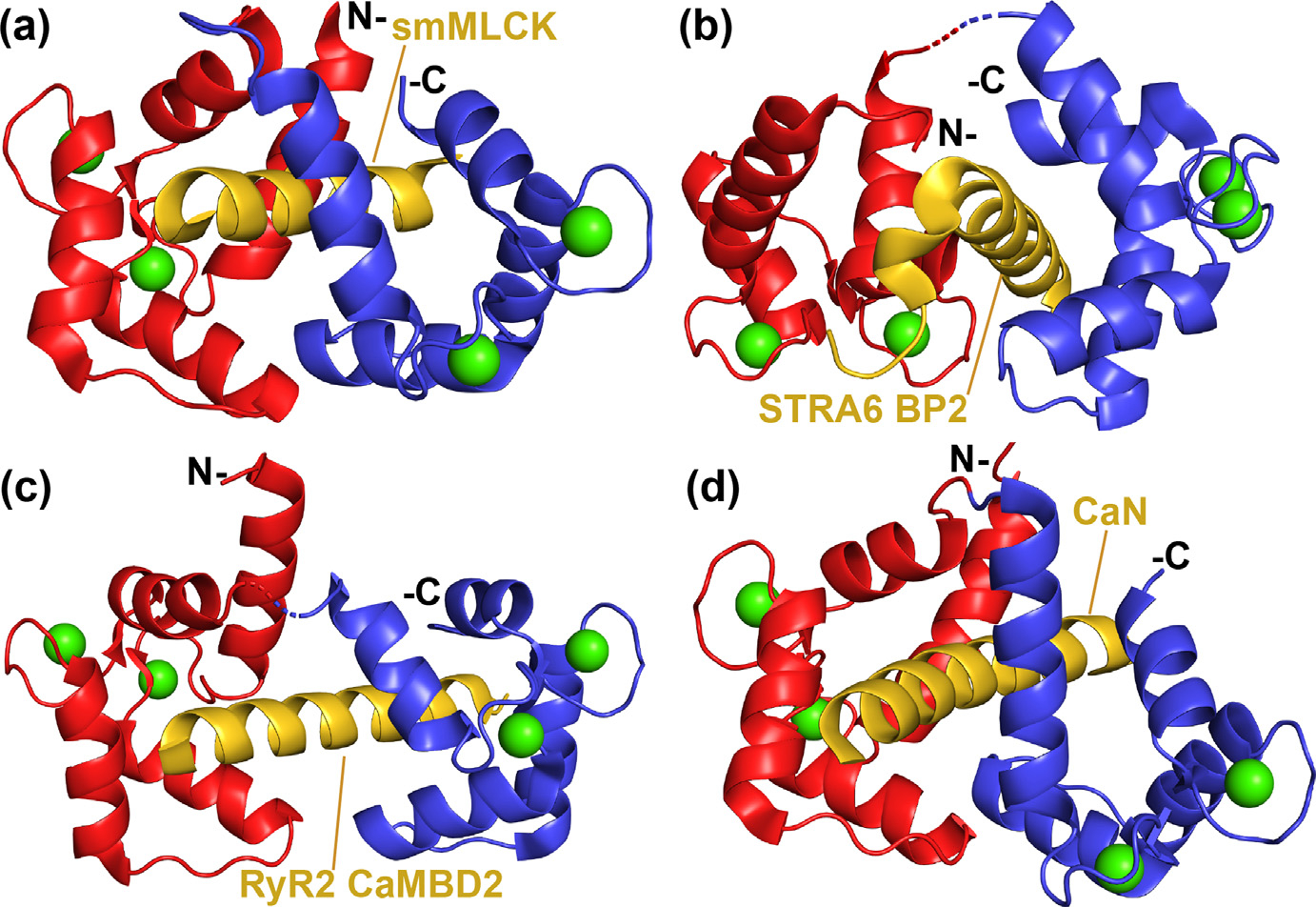
Binding modes for prototypical Ca^2+^-CaM target complexes. Ribbon diagrams of Ca^2+^-CaM bound to peptides derived from (A) smMLCK (PDBID: 2K0F),^232^ (B) STRA6 BP2 (PDBID: 5K8Q),^114^ (C) RyR2 CAMBD2 (PDBID: 6Y4O),^243^ and (D) CaN (PDBID: 4Q5U).^242^ In the presence of smMLCK, RyR2 CaMBD2 and CaN, ^Ca^CaM exhibits a “wrapping” binding mode where both domains of CaM (N-lobe, red; C-lobe, blue) surround the target alpha helix (orange). In contrast, hydrophobic residues of the C-lobe (blue) and just the opposite side of the N-lobe (red) interact with BP2 (orange) in non-canonical arrangement. Ca^2+^ ions are represented in all panels by green spheres.

Upon binding Ca^2+^, ^Ca^CaM also binds to a large diverse set of protein targets, such as serine/threonine kinases, membrane channels, transporters, and many others. For the most part such protein–protein interactions (PPIs) are dominated by a short alpha-helical segment of the target of ~20 residues termed canonical CaM-binding domains or regions (CaMBDs/CaMBRs), which are easily recognized by the spacing of bulky hydrophobic and basic amino acid residues (*i.e*., positions 1–10, 1–14, and 1–16).^161,164^ Unlike the IQ motif CaM targets, the affinity of CaM for Ca^2+^ typically increases via allostery upon target binding.^19,49–51,231–233^ For example, the tight binding of skeletal muscle myosin light chain kinase (skMLCK) peptide (^MLCK^K_D_=~1 nM) was reported to increase the affinity of CaM for Ca^2+^ by 100- and 160-fold in the N- and C-terminal domains, respectively (^Ca EF1/EF2^K_D_ = 80 ± 6 nM, ^Ca EF3/EF4^K^D^ = 20 ± 1 nM) (Table 3)^51,234^, and that activation allosteric mechanism proceeds via a “stepwise” Type 2 BFF mechanism^12–14^ (Figure 2). In many such CaM-target complexes, there is evidence that the C-lobe of CaM interacts with the target in the absence of Ca^2+^ altogether and/or at resting levels of Ca^2+^ (i.e., ~100 nM), but as Ca^2+^ levels increase upon a Ca^2+^-signal (Figures 1, 2), the N-lobe becomes occupied with Ca^2+^ ions. It is this second BFF event whereby ^Ca^CaM rearranges into an “active complex” in which two “foldon-like” N- and C-terminal domains of ^Ca^CaM wrap around the target protein (Figures 2, 7). Such a conformation is sufficient to induce a biological effect such as enzyme activation in the case the MLCK complex (Figure 1 (B)) or regulation of vitamin A transport for the STRA6 complex, as is outlined in detail else-where.^6,64,114,235^ Important to the stepwise mechanism is that ^Ca^CaM is associated with its biological target, even under resting conditions at ~100 nM, so the inactive CaM-target intermediate complex can efficiently respond to rising intracellular free Ca^2+^ upon a signaling event without having to recruit CaM to the site of the target. Such a scenario would be most important in cell-types where Ca^2+^ oscillations occur at a relatively high frequency such as in muscle and nerve.^236^

While the N- and C- domains of many canonical ^Ca^CaM are found to “wrap” around a target canonically, there are non-canonical arrangements of ^Ca^CaM-target complexes that can achieve a type 2 stepwise mechanism, including an increase Ca^2+^ ion binding affinity upon target-protein binding.^161^ Thus, the global orientations of the N-terminal and C-terminal domains, relative to the protein target, cannot alone be used to explain the allosteric effect. For example, a robust allosteric tightening of Ca^2+^ upon complex formation occurs for a non-canonical CaM complex involving the BP2 helix of the STRA6 vitamin A transporter,^64^ yet it is structurally unlike any other canonical ^Ca^CaM-target complex to date.^63–64^ However, upon closer inspection, the individual domains of CaM closely align with those of several CaM-target complexes and have similar EF-hand interhelical angles (I-II, III-IV, V-VI, VII-VIII) ranging within 15 of these other EF-hand interhelical angles (Table 3). These data provide insight that allostery involving the CaM family of CBPs is more global within the N- and C-lobes than via just their relative orientation within the CaM-target complex.

As discussed with S100 proteins, it is helpful to examine the dynamic properties of CaM upon the addition of Ca^2+^ and/or target to characterize CaM-target complexes and allostery.^124,127,219,249–250^ Most notably are NMR relaxation studies, which can measure backbone and sidechain motions of the CaM states in picosecond (ps) to > second (sec) timescales with atomic level detail under varying conditions.^213,251^ For example, the dynamics of the CaM backbone ^H^N correlations show that CaM exhibits both fast (ps – ns) and slow (μs – ms) time-scale motion in all four EF-hand domains in the absence of Ca^2+^ (EF1-EF4). Moreover, residues in the C-lobe were more dynamic compared to those in the N-lobe as indicated by larger number of backbone amide correlations exhibiting conformational exchange or by elevated rates of amide exchange as measured by H/D exchange.^249–250^ Low order parameters (S^2^), in the 0.5–0.6 range, were found also in the linker region (K77-S81) that connects the N- and C-lobes.^219,249–250^ Such experimental data was consistent with computational modeling in which Ca^2+^ binding to CaM stabilized the backbone dynamic properties of all four EF hand motifs as well as the EF hand connecting loops and linker region.^252^

In the presence of high affinity (nM) peptide targets that are comparable to full-length complex binding, such as CaM kinase I (CAMKI), CaM kinase kinase alpha (CaMKKα), and smMLCK, the backbone resonances of ^Ca^CaM rigidify internal motions even beyond that of adding Ca^2+^ to CaM.^124,126–127^ These data are fully consistent with allostery observed for Ca^2+^ binding upon ^Ca^CaM-target complex formation. In addition, a reduction in the dynamic properties of sidechain methyl resonances were observed for all three peptides derived from smMLCK, CAMKI and CaMKKα.^124,126^ It needs to be mentioned that decreases in both fast and slow time-scale motions of ^Ca^CaM in loops of the helix-loop-helix EF-hand domains, between helices II and III and VI and VII, respectively, and the linker region between the two domains, were observed upon target peptide binding. In summary, ^Ca^CaM is highly mobile in the absence of target, and these motions are reduced, throughout the entire CBP sequence in both the backbone and sidechain residues, and especially within the target binding interface.^124,126–127^ Moreover, it is thought that full-length targets would subdue the dynamic properties of ^Ca^CaM even more dramatically than peptide targets based on cryoEM structures of ^Ca^CaM bound to membrane-bound receptors such as STRA6 and RyR1 (Figure 6; however, as found for STRA6 peptides, these additional interactions, beyond the CaMBDs/CaMBRs are not a “driving” force for complex formation with ^Ca^CaM, but more likely to be relevant to inducing changes in the target protein as necessary for their biological function.^64^

It is important to consider kinetic components of type 2 stepwise mechanisms and how it impacts cellular function. In the case of CaM, many innovative methods were used for such purposes including some recent studies that combine stopped flow and magnetic resonance methods. This included a set of studies that monitored a coil to helix transition within the MLCK peptide upon ^Ca^CaM-MLCK peptide complex formation as a function of time.^12,253^ In one study the MLCK peptide was added to Ca^2+^-saturated CaM^253^; whereas in the other study addition of Ca^2+^ and the MLCK peptide were added simultaneously to apo-CaM.^12^ The latter experiments most closely mimicked a type 2 BFF mechanism for complex formation (Figures 2, 3) and showed that the N-terminal residues of the MLCK peptide bound to the C-terminal domain of ^Ca^CaM in two milliseconds (2 ms) followed by a second interaction between the C-termina residues of the MLCK peptide and the N-terminal lobe of ^Ca^CaM within eight milliseconds (8 ms) after mixing. These kinetic studies combined with paramagnetic relaxation enhancement (PRE) studies were used to characterize transient, compact, and sparsely populated states of CaM that were unable to be detected by traditional NMR methods and were in essence “invisible” by other structural methods. This PRE study demonstrated that a subset population of ^Ca^CaM, samples a range of compact structures along with the native, more extended structure, and that Ca^2+^ binding to CaM shifts the distribution of conformational states toward those that resemble the peptide bound state.^83 19^F relaxation measurements supported this mechanism of binding since conformational exchange was detected between a peptide-free native state of ^Ca^CaM and a state resembling the peptide-bound (near-native) state.^254^ Importantly, as mentioned, the MLCK peptide underwent a random coil to helix “folding” transition upon binding ^Ca^CaM in both studies, as is the case with several peptide targets when they bind to various S100 proteins.^12–14^ Moreover, the near-native state of the ^Ca^CaM-MLCK peptide complex is water-depleted in target-bound CaM near phenylalanine residues within the hydrophobic core, a property that is also recognized as being advantageous for binding a range of protein targets and consistent with “nucleation” events that are typically identified as driving “folding-like” changes in protein ensembles within a funneled energy landscape.^254^

In summary, for prototypical CaM-target complexes, CaM interacts with a target in a stepwise manner (Figures 2, 7). Step 1 in essence occurs at resting levels of Ca^2+^ (i.e., 100 nM), but this binding and folding event (BF) does not produce an active effector protein, but rather is on a functionally relevant pathway towards a functionally important complex. Step 2 represents the binding and functional folding event (BFF) and occurs as Ca^2+^ levels increase upon a Ca^2+^-signal (<300 nM; Figure 1). When this occurs the N-lobe becomes occupied with Ca^2+^ and the complex rearranges into what is a well-known “active form” of ^Ca^CaM-target complex in which two “foldon-like” domains of ^Ca^CaM, the N- and C-terminal domains, wrap around the target protein to induce a biological effect in its protein target (Figures 2, 7). Functions of CaM targets of this type can include enzyme activation, as is the case the MLCK complex described here or vitamin A transport for the STRA6 complex (Figure 3). Such a stepwise mechanism allows for CaM to respond efficiently to a Ca^2+^-signal inside the cell since it remains with its target protein prior to signaling, even at resting [Ca^2+^]^free^ of < 100 nM.

## Outlook

Cellular functions can be cycled “on” and “off” via changes in cytosolic [Ca^2+^]^free^ that occur within a narrow dynamic range of amplitude between 0.1 to ~1 micromolar. Ca^2+^-binding proteins (CBPs) serve as “sensors” of this Ca^2+^ current to deliver cellular function quickly by a physical action upon protein effectors (i.e., “on” switch), in a Ca^2+^-dependent manner. Upon Ca^2+^ dissociation from this CBP-effector complex, the functional response is ceased (i.e., “off” switch). Because specific cells require specific functions, [Ca^2+^], [CBP], and [effector protein] concentrations in their functionally relevant states are all tuned by the cell to provide and respond to cycles of basal [Ca^2+^]^free^ of ~100 nM in resting states and amplitude peaks of [Ca^2+^]^free^ of ~500 nM or sometimes higher in activated states. It is now typically recognized that those CBPs not involved in a target interaction also serve as a function to “buffer” free Ca^2+^ levels, so that they remain in the very low micromolar range. The genomic availability of a sufficient number of CBPs is needed to achieve this for each organism, and the CBPs within each cell must provide (i) the proper allosteric tightening of Ca^2+^ affinity needed to achieve such a strict dynamic range of [Ca^2+^]^free^ and provide the (ii) proper array of protein–protein interactions (PPIs) needed for specific cellular functions. As a mechanistic requirement for signaling, CBPs can only be saturated with Ca^2+^ when the target protein(s) and the elevated Ca^2+^ signal are available and each Ca^2+^-dependent CBP-effector complex needs to evolve so that it persists inside the cell for a time constant for optimal functionality (i.e., “on signal”), prior to Ca^2+^ dissociation (i.e., “off signal”).

It is likely that experimental tools and novel innovation for understanding allostery and many other aspects of protein functional dynamics will continue to lean on developing novel approaches including from NMR as well as expanded treatments of protein ensembles and energy landscape analyses^255–256^. Also, important will be advances in cryo-EM as well as tomography methods that can measure first-hand the relevant structural al details of proteins and protein complexes inside of a cell^257–260^. The rapid advancement of single particle imaging using X-ray laser pulses promises large libraries of structures from unfrozen samples within the next decade.^261–262^ Such data as they pertain to changes in states (i.e., Δstructure, Δdynamics on multiple time scales, Δchemical shift etc) and energetics throughout the protein complex will impact how we understand mechanisms for how specific complexes function. Such knowledge will also be guided by considering that such biomolecules were evolved for a variety of purposes/functions within a living cell and should be considered empirically for organizing such research via a BFF framework. Such an approach will most quickly allow us to apply this knowledge towards preventing and/or treating disease states.

## Summary

The calcium-ion (Ca^2+^) is key for biology and serves as a “second messenger” that delivers biological signals within a narrow dynamic range of intracellular free Ca^2+^ ion concentration, [Ca^2+^]^free^. For example, in muscle and nerve, cells are “resting” at ~100 nM of [Ca^2+^]^free^ but “active” at concentrations that approach ~500 nM (Figure 1). Moreover, gradients of [Ca^2+^]^free^ are distributed spatially and with cell-specific frequencies to deliver biological functions.^215,263^ Two subfamilies of EF-hand calcium-binding proteins (CBPs) are distinguished here for illustrative purposes since they represent two of many possible BFF types, one that is concerted (type 1; S100) and a second that is stepwise (type 2; CaM), respectively (Figures 2, 3). Although, many other distinct allosteric BFF types (BFFs) certainly exist in nature including those involving other second messengers, such as cAMP^264–266^ and cGMP.^267^ Studies of allostery with complexes involving these second messengers, or any biomolecule complex, could benefit by examination and/or reexamination within a BFF framework. Such a framework, as outlined here for CBP-target interactions should directly consider (i) the mass action of the exact components of a complex within the context of their location(s); (ii) the location and formation requirements of the complex to function; (iii) the lifetime of the complex and its time constant needed for function; and (iv) how it dissociates within a physiologically relevant timeframe to “turn off” the biological function, all of which are elegantly encoded within a physiological framework and by the physical chemistry that evolved for the biomolecular complex itself.

## Figures and Tables

**Table 1:** Dissociation constants of Ca^2+^ from the EF1 and EF2 domains of S100 proteins in the absence and presence of peptide targets (^Ca-EF1^K_D_; ^Ca-EF2^K_D_) are reported for various S100 proteins. Dissociation of target peptides from ^Ca^S100 proteins (^Target^K_D_) are also provided.

S100 protein (±peptide target)	^Ca-EF1^K_D_ (μM)	^Ca-EF2^K_D_ (μM)	Fold change in ^Ca EF2^K_D_ versus S100 alone	^Target^K_D_ (μM)

S100A1	250	27 ± 2^168^	-	-
S100A1 (+TRTK-12)	-	8 ± 3^169^	3.4	23 ± 6^169^
S100A1 (+RyR)	-	0.1^170^	270	8 ± 1^170^
S100A1 (+RIIβ)	-	0.3 ± 0.1	90	2 ± 1^5^
S100B	350	56 ± 9^171^	-	-
S100B (+p53)	-	20 ± 3^43,172^	>2.8	24 ± 7^171^
S100B (+TRTK12)	-	12 ± 10^171^	4.7	2.9 ± 0.5^171^
S100A4	>500	2.6 ± 1.2^173^	-	-
S100A4 (+p37)	-	0.2^174^	13	
S100A4 (+mysIIA)	3.6 ± 0.2^66^	0.26 ± 0.01^66^	10	‘<0.001^175^
S100A5	160	0.2^176^	-	-

**Table 2:** Ca^2+^-binding properties of CaM in the presence of peptide targets. Dissociation constants (K_D_ values) of CaM for Ca^2+^ from EF3/EF4 or EF1/EF2 in the presence of target peptide.

Target	IQ-motif	^Ca EF3/EF4^K_D_ (nM)	^Ca EF3/EF4^K_D_ Fold Change^a^	^Ca EF1/EF2^K_D_ (nM)	^Ca EF1/EF2^k_D_ Fold Change

None^51^	-	2,000 + 100	-	13,000 + 600	-
skMLCK^51^	NO	20 ± 1	100	80 ± 6	163
smMLCK^51^	NO	100 + 60	20	200 ± 100	65
BP2^64^	NO	60 ± 8	33	1,000 ± 100	13
iNOS^237^	NO	800	2.5	7,000	1.9
RyR2 CAMBD2^238^	NO	30	67	800	16
RyR2 CAMBD3^238^	NO	70	29	450	29
Neurogranin^227^	YES	2,000 ± 90	1	11,100 ± 120	1.2

a Fold change is ^Ca EF3/EF4^K_D_ for Ca^2+^ in absence of target (K_D_ = 2,000 nM) divided by the ^Ca EF3/EF4^K_D_ for Ca^2+^ in the presence of target.

b Fold change is ^Ca EF1/EF2^K_D_ for Ca^2+^ in absence of target (K_D_ = 13,000 nM) divided by the ^Ca EF1/EF2^K_D_ for Ca^2+^ in the presence of target.

**Table 3:** Interhelical angles within the CaM structure. Interhelical angles calculated by the program interhlx (K. Yap, University of Toronto), helices were defined by PDB coordinates.^246^ Rotation angle of bending residues was determined to occur between 130–180° DynDom^247^ for ^Ca^CaM-peptide complexes compared to ^Ca^CaM-BP2. DynDom determined the axis of rotation to be the linker region.

Protein/Peptide Target	Ca^2+^-bound	PDBID	Interhelical Angles (°) (Ω)			
			I-II	III-IV	V-VI	VII-VIII

None^b^	NO	1DMO^239^	131	132	137	133
IQ-motif Na_v_1.5	NO	2L53^240^	127	127	108	123
Na_v_1.4C-Terminal Domain	NO	6MBA^229^	135	134	105	114
Nav1.2 IQ	NO	6BUT^228^	131	132	113	111
None^b^	YES	1CLL^241^	89	87	102	95
STRA6 BP2^a^	YES	5K8Q^114^	94	96	99	104
Smooth muscle myosin light chain kinase^a^	YES	1CDL^241^	89	86	97	98
Calcineurin (CaN) ^a^	YES	4Q5U^242^	92	83	107	103
Ryanodine receptor 2 (RyR2) CAMBD2 ^a^	YES	6Y4O^243^	83	82	103	98
NMDA Receptor NR1 C1 ^a^	YES	2HQW^244^	92	85	91	89
Voltage-gated sodium channel Na_V_1.4 IQ ^a^	YES	6MUE^245^	108	94	99	93

a Reported by Young et al..^64^

b From https://structbio.vanderbilt.edu/cabp_database/struct/ih_ang/calmod_helang.html, a Ca^2+^ binding protein data library.

**Table 4:** Peptide-binding properties of Apo- and Ca^2+^-CaM. Dissociation constants (K_D_ values) of CaM binding to target peptides in the absence (^Apo^CaM; ^Target^K_D_) and presence (^Ca^CaM; ^Target^K_D_) of Ca^2+^.

Target	IQ-motif	^Apo^CaM -^Target^K_D_ (nM)	^Ca^CaM- ^Target^K_D_ (nM)

skMLCK^51^	NO	-	1
smMLCK^51^	NO	-	1–2
BP2^64^	NO	140 ± 30	0.9 ± 0.3
iNOS^237^	NO	40–100	3.3
NMDA NR1 C0^225^	NO	2,250	87
NMDA NR1 C1^244^	NO	158 ± 3	2.0 ± 0.1
RyR2 CAMBD2^248^	NO	390	1
RyR2 CAMBD3^248^	NO	530	7
Na_V_1.2^228^	YES	6.3	19.8
Na_V_1.4^229^	YES	17 ± 3	280 ± 30
Na_V_1.5^230^	YES	160	2,000
Neurogranin^227^	YES	205	300

## Abbreviations:

CBPs: Calcium-binding proteins
CaM: calmodulin
F: Folding
BF: Binding and folding
BFF: Binding and functional folding
PPI: protein-protein interaction
IDPs: intrinsically disordered proteins
R_ex_: chemical exchange parameter
MM: malignant melanoma
CaMBDs/CAMBRs: CaM-binding domains or regions
RyR: Ryanodine receptor
CaN: Calcineurin
smMLCK: smooth myosin light chain kinase
skMLCK: skeletal muscle myosin light chain kinase
BP2: STRA6 binding peptide 2
PKA: protein kinase A
NMIIA: nonmuscle myosin IIA
S^2^: general order parameter
CAMKI: CaM kinase I
CaMKKα: CaM kinase kinase alpha
PRE: paramagnetic relaxation enhancement
